# How to parameterise an equivalent-circuit empirical battery model from time-domain data

**DOI:** 10.1016/j.mex.2026.103865

**Published:** 2026-03-14

**Authors:** Mark Blyth, Amey Gupta, Alastair Hales

**Affiliations:** a University of Bristol, UK; b The Faraday Institution, UK

**Keywords:** Lithium ion, Sodium ion, Equivalent circuit, ECM, Parameter identification, Battery modelling

## Abstract

Empirical battery models have many uses, including pack design, charge estimation, and cell selection. An accurate model requires accurate parameters, however there are a range of subtleties in parameter fitting that are often missed in the literature. This work presents a best-practice guide for parameterisation, covering design of appropriate experiments; using numerical optimisers to fit model parameters to the experimental data; and building a suitable interpolation of the fitted model parameters, to get the required parameter-functions for a model. Key novelties include the importance of using timescales in place of capacitances for numerical stability; the design of a multi-step parameterisation process where model complexity is gradually increased to give more accurate parameter estimates; and a combined numerical and experimental design, so that the right experiments, data, and fitting methods are chosen for the target application. Our method is presented as a general recipe, so that users can easily choose the right setup for their needs. Scripts are provided which implement the method and reproduce the provided examples. Additionally, a full set of experimental data, model scripts, and fitted parameters are provided, both for testing the described method and for re-use in modelling studies.

## Graphical abstract

**Figure:**
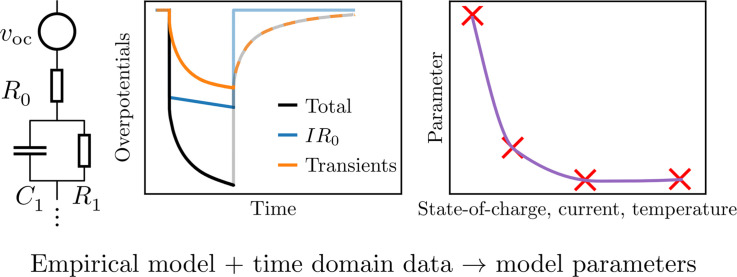

## Background

Parameterisation involves finding the model parameter values that give the best possible match between model predictions and experimental data. Various types of battery model exist, and different parameterisation methods are needed for each model type. Electrochemical models describe the underlying physics inside a cell, which gives rich information about internal cell states [14]. These can be used for questions such as cell degradation [1], and to assess how cell design impacts current flows, heat generation, and overall performance [2]. Electrochemical models are powerful, and can be combined with data-driven models [16], however they are expensive to parameterise and often too computationally complex for use in battery management or pack design. Empirical models such as equivalent circuits are cheaper to parameterise, and computationally simpler. They aim to reproduce cell behaviours without describing where they come from. This is beneficial for engineering-scale problems such as battery management [3]. Empirical models can also generalise well to the heterogeneous conditions seen in large-format cells and batteries [4], and thus retain good voltage accuracy outside the lab.

Electrochemical models use teardown parameterization, which involves dismantling a cell to measure properties such as electrode thickness or particle size. Alternatively, representative parameters can be taken from the literature when similar cells have been studied before [5]. Frequency-domain parameterisation is common for both empirical and electrochemical models, whereby a small alternating current is used to find cell impedance, and processed to extract model parameters [6]. Finally, time-domain parameterisation is common for cell and pack scale empirical models. A current stimulates a cell, and numerical methods are used to identify model parameters from the measured voltage response. Engineering problems usually use empirical models and time-domain parameterisation.

Various researchers have either developed or used time-domain parameterisation methods and empirical models [[7], [8], [9], [10]]. Parameterisation can be applied as part of state [17], and efficient numerical methods have been developed for parameterising during cell relaxation [18]. Despite these developments, improvements can be made to yield more accurate cell models. Our method includes several novel contributions which help in getting the best parameters. Our multi-step parameterisation gradually increases the model complexity, so that the changeability in series resistance is captured during later parameterisation steps, which improves the overall model accuracy. Cells behave differently at rest or under load, and at different current draws, temperatures, and states-of-charge, so the parameterisation data are chosen around this to maximise accuracy. We show that parameterising with data from a resting cell causes a reduction in model accuracy. Recent works have highlighted the impacts experimental data can have on model accuracy [19], however practical identifiability - the ability to extract parameters from real-world data - is still regularly overlooked in the battery parameterisation literature. This method pays careful attention to identifiability, by designing the experiments, model structure, and numerical methods around what can be meaningfully found from data.

This work provides a fully general framework, which explains how to parameterise a new cell or battery model, and provides guidance on the tradeoffs and design choices that need to be addressed when doing so. It is based on an approach that has been gradually developed through multiple projects, covering numerous cell form factors and several cell chemistries, and has fed into refs [3,4]. We provide a detailed overview of the parameterisation method design, and explain how it can be applied to new models. The method is designed to be fully compatible with modern computational and software tools, such as PyBOP (Python Battery Optimisation and Parameterisation) [11] and PyPROBE (Python PROcessing for Battery Experiments) [12]. Example scripts have been provided, both for applying the techniques described here, and to generate all the results shown in this work. The work constitutes a best-practice guide, by addressing common literature pitfalls in a generalisable and easy to use setup.

## Method details

The method proceeds by first collecting experimental data, choosing a model structure, then sequentially fitting model parameters. Cell capacity is determined from experimental data, which is then used to find the state-of-charge throughout a current-pulse test. Open-circuit voltage curves are extracted, which then allows model parameters to be identified through a multi-step least-squares fitting procedure. Finally, interpolation is used to create functional parameters from the identified samples. Novelty comes from the robust numerical design; co-creation of experimental profiles, numerical methods, and model applications to maximise practical identifiability, including only fitting to data when a cell is under load; and from the multi-step parameterisation approach.

Parameterisation involves matching a model to experimental data, and so, Section 2.1 begins by introducing the model. We present the method as a step-by-step recipe, so that the general recipe can be tuned to the cell of interest. This relies on a distinction between the parameters of the model, which we aim to fit; and parameters of the fitting procedure that the user must choose before starting experiments and parameter extraction, which we call hyperparameters. Section 2.2 explains how to choose hyperparameters, then Section 2.3 outlines the fitting procedure itself. Common issues are highlighted throughout the text. Scripts implementing the method are provided alongside a model, experimental data, and fitted parameters, and can be accessed on GitHub at https://github.com/MarkBlyth/parameterisation_methodsx, and Zenodo at https://doi.org/10.5281/zenodo.19072964.

### Model overview

**Fig. 1:**
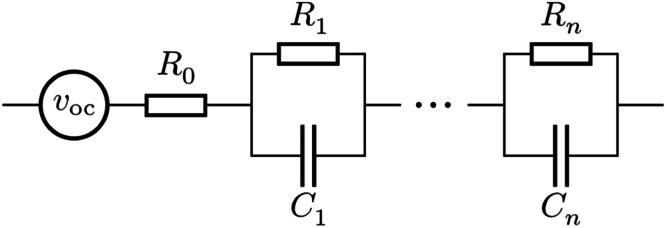
Circuit representation of the Thevenin battery model. Parameterisation builds up functions to describe how each circuit element depends on temperature, current, and state-of-charge.

A variety of empirical battery models exist [13]; this method focuses on the Thevenin model, often represented as the circuit model shown in Fig. 1, as it is the simplest model structure that can capture overpotentials, transients, and state-of-charge dependency on the open-circuit voltage. An ideal voltage source $v_{\text{oc}}$ provides the cell potential. Cells have an internal resistance, meaning the voltage under load differs from the open-circuit voltage; these effects are partially captured by series resistance $R_{0}$. Additionally, mass transport effects cause transient changes to the cell voltage, which are captured by the parallel resistor-capacitor (RC) pairs. Resistances and capacitances of each component will vary with temperature, state-of-charge, and current I. Eqs. (1) therefore expresses them as functional parameters. The presented method can be adapted easily for constant parameters, or parameters that depend on one or more of T, SOC, and I.

The full model links $v_{\text{batt}}$ [V], the output voltage of a cell or battery, to state-of-charge SOC and transient voltages $v_{rc_{i}}$ [V], for i=1,…,n, and is commonly expressed as

(1) $$\left\{ \begin{array}{cc} \frac{d}{dt}\mathrm{SOC} & =\frac{I\left(t\right)}{3600\;Q_{\text{nom}}},\\ \frac{dv_{rc_{i}}}{dt} & =\frac{1}{C_{i}\left(T,\mathrm{SOC},I\right)}\left(I\left(t\right)-\frac{v_{rc_{i}}}{R_{i}\left(T,\mathrm{SOC},I\right)}\right),\\ v_{\text{batt}}\left(t\right) & =v_{\text{oc}}\left(T,\mathrm{SOC}\right)+I\left(t\right)R_{0}\left(T,\mathrm{SOC},I\right)+\sum_{i=1}^{n}v_{rc_{i}}, \end{array}\right.$$

where open-circuit voltage $v_{\text{oc}}$ [V], resistances $R_{i}\;[\Omega$], and capacitances $C_{i}$ [F] are all functions of temperature T [C] and state-of-charge SOC, and optionally, current. I(t) [A] is the current into or out of the cell and taken as positive during charge. A scale-factor of 3600 is used in the state-of-charge equation, so that nominal capacity $Q_{\text{nom}}$ has units Amp-hours and time t has units seconds.

• ***Common issue:*** differing sign conventions for I(t).
• ***Solution:*** check sign conventions across the toolchain, and ensure they are consistent. Sign conventions can differ between model equations, modelling software, and battery cyclers; some take current as positive in discharge, and some take it as negative. Either approach is valid, but must be used consistently.

Resistors and capacitors govern the rate that transient voltages change. Transient changes follow a timescale τ, given by $\tau_{i}=R_{i}C_{i}$ [s]. The transients equation can be written in terms of these timescales, as

(2) $$\tau_{i}\frac{dv_{rc_{i}}}{dt}=IR_{i}-v_{rc_{i}},$$

which shows how RC voltages tend towards a voltage of $IR_{i}$ with timescale $\tau_{i}$. Resistor-capacitor pairs are ordered by $\tau_{1}<\tau_{2}<...$, so that $\tau_{1}$, being smallest, describes the quickest transient behaviours.

It is useful to work with timescales instead of capacitances. Capacitance parameters are often many times bigger than resistances, which can cause difficulties for an optimisation algorithm. Using a timescale instead of a capacitance improves parameterisation performance by reducing the difference in scale of each parameter.^1^ Additionally, optimisation problems are fairly insensitive to the capacitance value - $C_{i}$ can be changed large amounts without significantly impacting the model fit. Problems are more sensitive to timescales $\tau_{i}$, so an optimiser is more likely to identify good parameters when the model is expressed in timescales. The resulting models are also more numerically stable. Parameterisation routines generate a set of parameter samples, which are then interpolated for use in a model. For interpolants R and C, their product τ=RC can behave unexpectedly between datapoints, and may vary substantially even when R and C are individually sensible. This can lead to large model errors, which are avoided if the timescales themselves are used to build an interpolation.

• ***Common issue:*** using capacitances $C_{i}$ when modelling and parameterising; this makes it more difficult to find good parameters, and more likely for the model to become unstable.
• ***Solution:*** parameterise and model with timescales $\tau_{i}=R_{i}C_{i}$ instead of capacitances $C_{i}$, using Eq. (2).

#### Choosing recipe hyperparameters

For parameterisation to succeed, the numerical and experimental setups must be designed properly. These are described by a set of design parameters which we refer to as hyperparameters. This section provides an overview of what each hyperparameter is, and how they should be chosen. We generally avoid recommending specific hyperparameter values, because they will depend heavily on the test-cell and the model usage-case; however, Section 3 provides an example of the method being used, and lists the hyperparameters that were chosen.

#### Test temperatures $T_{\text{test}}$

Parameterisation tests should be conducted across the full temperature range of interest. Subject to safety limits, the lowest tested temperature should be just below the coldest conditions a cell model will see, and the highest tested temperature should be just above the hottest conditions. Cell parameters change more rapidly at lower temperatures, and therefore it is reasonable to run fewer experiments at high temperatures. A typical test suite might be 25 ${}^{\circ }$C, 40 ${}^{\circ }$C, 15 ${}^{\circ }$C, 10 ${}^{\circ }$C, 5 ${}^{\circ }$C. Ordering the tests like this means lower-temperature experiments, which are more likely to degrade the cell, are kept until last.

#### Pulse current $I_{\text{pulse}}$

**Fig. 2:**
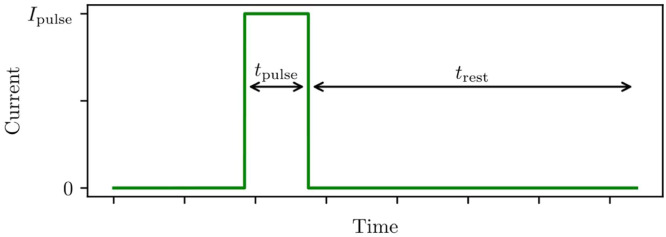
Profile for a single current pulse. Current is switched back on immediately after the rest-time $t_{rest}$. Pulsing continues until the cell reaches upper or lower voltage cutoff.

See Fig. 2 for an illustration of pulse current. Experimental data are collected by applying current pulses, and measuring the voltage response. The current of each pulse should be matched to the target use-case of the battery model. For example, a model for fast-charge should be parameterised with high currents, and a model for trickle-charging should use low currents. If necessary, tests and parameterisation can be performed with a range of current values. High-performance thermal control becomes increasingly important for larger currents.

#### **Settle-time**$t_{\text{rest}}$

See Fig. 2 for an illustration of settle-time. Settle-time controls how long a cell is left to rest after each current pulse. It must be long enough that the cell voltage stops changing. This ensures open-circuit voltage $v_{\text{oc}}$ is accurate, and also guarantees the initial conditions $v_{rc_{i}}=0$ are satisfied at the start of each pulse. The time this takes will vary across the state-of-charge range, and $t_{\text{rest}}$ must be long enough for every pulse to have settled. Trial and error can be used to choose $t_{\text{rest}}$, however this can take a long time. As a better alternative, some battery cyclers allow the resting step to be terminated when the voltage change dv/dt falls below some small threshold tolerance. This method is preferred as it removes the need for trial and error, and reduces overall experiment time by keeping the settle-time of every pulse as short as possible.

#### Timescale bounds $\tau_{i}^{\text{min}}$**and**$\tau_{i}^{\text{max}}$

Constraints need to be used when fitting for timescale parameters $\tau_{i}$. As an example, when fitting $R_{1}$ and $\tau_{1}$, a result of $\tau_{1}=1$ second would not be accurate or meaningful if it came from data sampled every 5 s. Similarly, $R_{2}$ and $\tau_{2}$ will be meaningless if, say, a parameter of $\tau_{2}=120$ seconds has been fitted using 10 s of data. In both cases, the data do not contain enough information to accurately identify the parameters. Timescale constraints are used to avoid this, which ensures that any identified parameters are reasonable.

The fastest timescale $\tau_{1}$ is constrained by the sampling rate of the experimental data. A rule of thumb is to take a lower-bound of $\tau_{1}^{\text{min}}=10\;t_{\text{sample}}$, where data are sampled every $t_{\text{sample}}$ seconds. For example, a sample rate of 1 Hz corresponds to $t_{\text{sample}}=1$, and a constraint of $\tau_{1}^{\text{min}}=10$ s gives 10 datapoints across the span of the first time constant.

A similar rationale is used for choosing the timescale upper bounds. It is not possible to accurately fit a transient if it barely changes throughout the available data range. Hence, $\tau_{n}^{\text{max}}$ should be chosen alongside the pulse-time $t_{\text{pulse}}$, so that a model does not fit timescales that last longer than the available data. A rule of thumb is $\tau_{n}^{\text{max}}=t_{\text{pulse}}$.

Generally speaking, the same bounds $\tau_{n}^{\text{max}}$ can be used for each timescale, so $10\;t_{\text{sample}}\leq \tau_{i}\leq t_{\text{pulse}}$ are a useful starting point. Nevertheless, it can sometimes be helpful to guide the optimisation by tuning each lower and upper bound around the expected timescales in the data. When doing so, it is important that each window overlaps substantially ($\tau_{i}^{\text{max}}>\tau_{i+1}^{\text{min}}$), to avoid artificially clipping timescales to the constraint boundaries. Bounds-tuning is best avoided, as better choices of initial conditions will almost always lead to better results; the key exception is when the optimal parameters lie outside the chosen bound range.

#### Pulse time $t_{\text{pulse}}$

See Fig. 2 for an illustration of pulse time. The time-span of each current pulse is an important parameter, and should be chosen with great care. Pulse time controls how much the state-of-charge changes through each pulse. Shorter pulses mean the state-of-charge and cell temperature will change, so that each parameter will be more accurate. Accuracy is further improved since more parameter samples are available across the full state-of-charge range. However, slow timescales cannot be identified from fast pulses, so each pulse must be long enough to capture all the transients of interest. It is essential that $t_{\text{pulse}}$ is chosen around $\tau_{n}^{\text{max}}$, the slowest transient-timescale a cell exhibits. Since $\tau_{n}^{\text{max}}$ will not be known ahead of time, an element of trial and error or domain knowledge is required. The authors typically use pulse times of two to six minutes, with preference towards shorter pulses, however this will depend both on the chosen cell, the pulse current, and the model application.

#### Sample rate $t_{\text{sample}}$

Choice of battery cycler sampling rate is important, as it determines the fastest transients that can be found in the data. A higher sample rate allows faster transients to be resolved, though this is only beneficial if the model application needs to resolve fast dynamics. The only drawbacks of high sample rates are larger data-files and slower numerical optimisation. Therefore, it is reasonable to use the fastest available sampling rate, with the option of downsampling data later. Alternatively, if the fastest timescales of interest are already known, sample rates can be chosen as 20 times faster than this timescale, based on the $\tau_{1}^{\text{min}}=10\;t_{\text{sample}}$ rule-of-thumb introduced in the timescale-bounds section, plus a safety margin.

As with timescale bounds, sample rate will be determined by model usage. For example, a model that updates a state estimate once a second does not need millisecond accuracy; the model needs to be accurate down to one second, so a fastest timescale of 1 s is appropriate, and a sample rate of between 10 Hz and 20 Hz can be used.

#### Δt**: Time-window for fitting**$R_{0}$

A multi-step fitting procedure is proposed, where series resistance $R_{0}$ is fitted before the transient parameters $R_{i}$ and $\tau_{i}$, then used as a functional parameter for later parameterisation steps. Step 5 fits a series resistance $R_{0}$ using only data from the first Δt seconds of a pulse. This has two benefits. Firstly, using a short time window reduces how much $R_{0}$ changes due to state-of-charge. Secondly, using a shorter span of data means that only the faster timescales need considering. Slower timescales can be ignored over short periods, so that a simpler model can be used for the $R_{0}$ fitting step. This improves the overall parameter fitting accuracy. Time window Δt should comparable to the fastest timescale $\tau_{1}$; somewhere in the range $\tau_{1}<\Delta t<2\tau_{1}$ is generally suitable. While $\tau_{1}$ is not known ahead of time, an estimate can be made, or trial and error can be performed. This does not need to be chosen too carefully, as a range of values will lead to the same $R_{0}$. Note that this can be omitted when $R_{0}$ changes slowly with state-of-charge.

#### Number of model RC pairs, n

Voltage transients come from the build-up and release of concentration gradients in electrode particles and electrolyte. An empirical model approximates the transients using a sum of exponentials, corresponding to the resistor-capacitor branches in the circuit model. It is worth noting that this is just an approximation, and the exponential terms do not correspond to specific cell processes. Consequently, the number of RC pairs needed is not universal, and will depend on the cell being tested. Choice of n should balance model accuracy against simplicity. Complex models with large numbers of RC pairs are liable to overfit, whereas simple models with a single RC pair will often give a poor approximation of the data. Bayesian methods can be used to automatically balance complexity and accuracy [21]. Alternatively, trial and error may be used. If an existing model fails to capture a large amount of transients, the parameterisation setup should be explored first, by checking the parameter bounds, optimiser choice, and initial parameter guesses. If the model is still underperforming, a further RC pair can be tried.

The available data also influence the number of RC pairs that can be used. A typical experiment might use sample rates $t_{\text{sample}}$ between 1 Hz and 0.1 Hz, pulse lengths $t_{\text{pulse}}$ of two to three minutes, and n=2 RC pairs. High sample-rates allow fast dynamics to be resolved, so that an additional RC pair can be included to model the faster dynamics; similarly, a longer pulse can allow for an additional RC pair to capture slower transients.

Importantly, equivalent circuits are empirical and will never provide a perfect fit, so simple models should be preferred to avoid overfitting. As such, it is preferable to keep n small. Values above two or three are rarely useful.

### Method recipe

The cell being parameterised is secured in a test rig to connect it to a battery cycler. A good test rig should have a low resistance at all electrical contacts, and independent current-carrying and voltage-sense cables to offset unwanted resistances. A battery cycler controls current to the cell, performs safety monitoring, and records the applied current and resulting voltage responses. Voltage responses are measured between the positive and negative cell terminals, using the voltage-sense cables. A thermal control strategy should be used for maintaining cell temperature, such as a climate chamber or surface cooling setup. Temperature should be monitored throughout the experiment, both for safety monitoring and to assess the cell temperature changes.

Experimental data are collected using the Galvanostatic intermittent titration technique. Galvanostatic means current is controlled and voltage is measured; intermittent titrations mean the electrochemical reaction is run (a titration is performed) for short periods (intermittently). Intermittent current pulses show both the instantaneous and transient changes in cell voltage. These can then be used to identify the various model parameters. Intermittent pulses keep temperature changes to a minimum, which reduces error in the parameter estimates. Steps are as follows.

#### Step 1: Collect data

##### Method

Data are collected by performing pulse-discharge and pulse-charge experiments at a range of cell temperatures.

1. Set cell to 25 ${}^{\circ }$C.
2. Charge cell to 100 % state-of-charge, using manufacturer-recommended procedures.
3. Set cell to test temperature $T_{\text{test}}$.
4. Pause for $t_{\text{pause}}$.
5. Discharge at current $I_{\text{pulse}}$ for time $t_{\text{pulse}}$.
6 Repeat, alternating between discharge-pulses and pauses until lower voltage cutoff is reached.
7 Pause for $t_{\text{pause}}$.
8 Repeat in charge: apply alternating charge-pulses and pauses, until upper voltage cutoff is reached.
  • ***Common issue:*** experiment designs such as pulse time, rest time, and sample rate need to be matched to the behaviours of the cell and the planned use-case of the final model.
  • ***Solution:*** the experimental data will govern what the model can or cannot describe. Experiments should be designed as explained in Section 2.2, so that the current pulses capture all the transients of interest, sample rates are sufficient to measure the transients of interest, and the cell has enough rest time to reach open-circuit after each current pulse.

##### Hyperparameters

• $I_{\text{pulse}}$ [A]: current used for each titration.
• $T_{\text{test}}\;[$C]: temperature of a given test.
• $t_{\text{pulse}}$ [s]: length of each pulse
• $t_{\text{rest}}$ [s]: time between pulses.

These hyperparameters are sketched in Fig. 2.

##### Explanation

The cell should always be recharged before testing, so that the state-of-charge is known accurately at the start of each procedure. Importantly, open-circuit voltage changes slightly with temperature, so the capacity stored after a recharge will depend on the charging temperature. Cells should therefore always be recharged at a standardised temperature, so that ‘fully charged’ is consistent across tests. We suggest 25 ${}^{\circ }$C to reduce cell degradation, however any consistent choice is suitable.

Current draws reveal the kinetic behaviours of a cell, and rests allow it to settle back to steady-state. Cell voltage will change for a while after the current has been applied, and rest-time $t_{\text{pause}}$ allows the voltage to settle. This enables $v_{\text{oc}}$ to be determined in step 2, and defines the model initial conditions for steps 3, 5, and 6. The combination of resting before each pulse, and a standardised state-of-charge reset, ensures the initial conditions for Eqs [eq:lumped_model]. are known. At the start of the experiment, SOC=1 from the state-of-charge reset, and $v_{oc_{i}}=0$ since the cell has rested until transients decay to zero.

To fully explore the state-of-charge range during charging pulses, cells could be held at lower voltage cutoff using a voltage hold, after the discharge pulses are finished. Nevertheless, the very bottom of the state-of-charge range is not usually accessed in normal usage, so engineering problems do not typically need to model this region.

#### **Step 2: Find nominal cell capacity**$Q_{\text{nom}}$

##### Method

Cell capacity is found by seeking the greatest achieved capacity throughput.

**Fig. 3:**
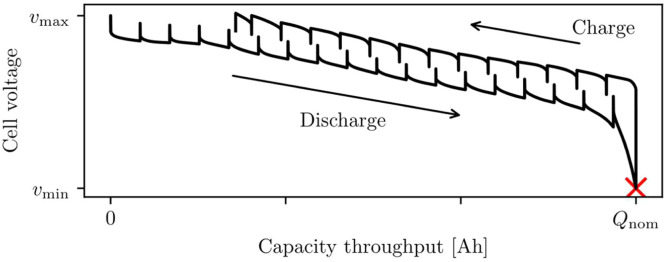
Capacity throughput is found by integrating current with respect to time, and nominal capacity is taken as the highest capacity passed through a cell.

1. Take the experimental data from step 1 (or a pseudo-open-circuit voltage sweep, if available).
2. Choose the dataset corresponding to the hottest temperature $T_{\text{test}}$, and lowest current $I_{\text{pulse}}$.
3. Apply Coulomb counting to find the capacity throughput Q(t) at any given time t in the experiment.
4. Take nominal capacity $Q_{\text{nom}}=\text{max}\left[Q(t)\right]$ as the maximum capacity throughput achieved in the experiment (sketched in Fig. 3).

Coulomb counting is performed by integrating the current I(t) with respect to time t [s]. This is expressed as

$$\frac{d}{dt}Q\left(t\right)=\frac{I\left(t\right)}{3600},$$

or equivalently,

$$Q\left(t\right)=\int_{0}^{t}\frac{I\left(\tilde{t}\right)}{3600}\;d\tilde{t}\;.$$

Capacity throughput Q(t) [Ah] is taken as zero at the start of the test, Q(0)=0. This step takes current as positive in discharge, so that Q(t) and the resulting cell capacity is positive. The scale-factor of 3600 is used because data are typically recorded in seconds, whereas capacity is conventionally measured in Amp-hours. Numerical integration should be performed using the trapezium rule.

##### Explanation

It is useful to have a consistent method for defining state-of-charge, in order to compare cells and define C-rates. An accurate nominal capacity also means state-of-charge will behave as expected, with SOC=0 when fully discharged and SOC=1 after a full charge. Since the exact capacity will vary with the age of a cell, it is useful to complement manufacturer datasheets with a standardised method for estimating capacity. Furthermore, not all cell capacity will be accessible due to the internal resistance of a cell, so there is an additional benefit in defining the nominal capacity around the testing conditions of interest. Nevertheless, chasing an extremely accurate nominal capacity is not necessary for building an accurate model, and datasheet values are often sufficient.

Nominal capacity should represent the most capacity that can be accessed from a cell. Since tests start at SOC=1, the point of highest capacity throughput gives the capacity of the cell. Small overpotentials are needed to extract the most capacity. Smaller currents and higher temperatures (lower cell resistance) give a lower overpotential, so the hottest, lowest-current experimental dataset should be used when determining cell capacity.

Trapezium rule integration is sufficient for the Coulomb counting step. Current is constant throughout a pulse, so trapezium rule numerics will not suffer from truncation errors. Cumulative summation can also be adequate, provided the battery cycler switches current sufficiently quickly.

#### **Step 3: Find state-of-charge**SOC(t)

##### Prerequisite parameters

• $Q_{\text{nom}}$: nominal capacity from step 2.

##### Method

State-of-charge is found by Coulomb-counting with the nominal capacity of a cell.

**Fig. 4:**
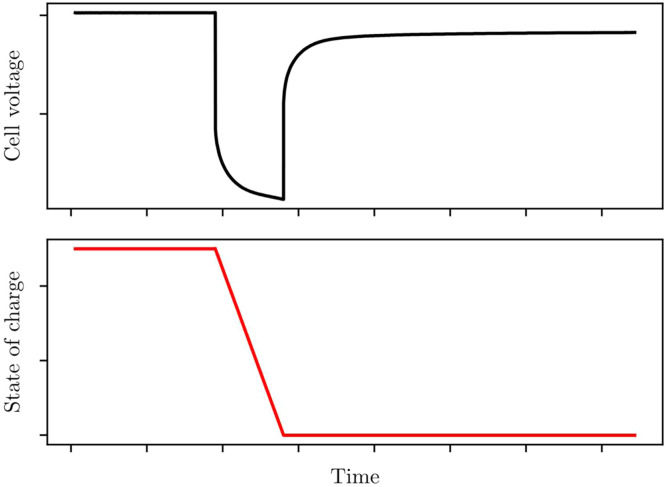
State-of-charge is found by integrating current throughput. Calculated state-of-charge is used instead of time for the independent variable.

1. Take the experimental data from step 1.
2. Take the nominal capacity $Q_{\text{nom}}$ from step 2.
3. Apply Coulomb counting, to find the state-of-charge at any given time t in the experiment, shown in Fig. 4.

Previously, Coulomb counting was performed by integrating the current I(t) with respect to time t, to get capacity throughput. State-of-charge is found by scaling this with respect to nominal capacity, expressed as

$$\frac{d}{dt}SOC\left(t\right)=\frac{I\left(t\right)}{3600\;Q_{\text{nom}}},$$

or equivalently,

$$SOC\left(t\right)=\int_{0}^{t}\frac{I\left(\tilde{t}\right)}{3600\;Q_{\text{nom}}}\;d\tilde{t},$$

with

SOC(0)=1.

State-of-charge is taken as 1 at the start of the test, due to the state-of-charge reset at the beginning of each experiment. Current I(t) is measured in Amps, capacity $Q_{\text{nom}}$ in Amp-hours, and time in seconds. As before, numerical integration should be performed using the trapezium rule.

##### Explanation

Model parameters $v_{\text{oc}}$, $R_{i}$, and $\tau_{i}$ all depend on state-of-charge, so SOC must be found so it can be used as the dependent variable for each parameter.

#### **Step 4: Find open-circuit voltage**$v_{\text{oc}}$

##### Method

Each pulse-cycle consists of a current pulse and a relaxation back to open-circuit voltage. The voltage at the end of each relaxation is sought.

1. Take the final voltage measurement from each pulse-cycle (when the cell has is at its most rested).
2. Record the open-circuit voltage and state-of-charge pairs $\left(SOC,v_{\text{oc}}\right)$ for each discharge pulse, as per Fig. 5.

**Fig. 5:**
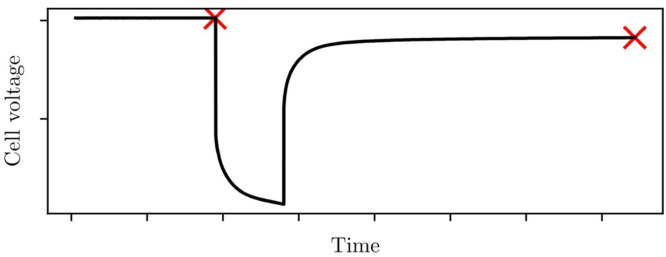
Open-circuit voltage is found from the voltage the cell settles to at the end of each pulse-cycle, which is marked here with crosses. Open-circuit voltage is recorded alongside the state-of-charge at that point, giving samples $\left(SOC,v_{OC}\right)$. This is repeated across all the pulses in discharge, then charge.
3. Repeat for each charge pulse.
4. Repeat for each tested cell temperature.
5. Use the samples $\left(SOC,v_{\text{oc}}\right)$ to build separate interpolation functions for $v_{\text{oc}}\left(SOC,T\right)$ in discharge and charge; see Step 7 for details on interpolation methods.

##### Explanation

Taking the voltage at the end of the resting time ensures the terminal voltage is as close as possible to open-circuit.

Separate discharge and charge datasets should be collected for each temperature. Open-circuit voltage will change with temperature due to entropic effects, so separate datasets should be compiled for each experimental cell temperature. Likewise, open-circuit voltages can vary between charge and discharge due to hysteresis, so separate datasets should be generated for the charge and discharge regimes. Note that methods exist for parameterising the entropy effects in open-circuit voltage [15], and for parameterising hysteresis models [3].

Interpolation methods for $v_{\text{oc}}\left(SOC,T\right)$ are considered shortly, alongside those for $R_{i}$ and $\tau_{i}$.

#### **Step 5: Fit**$R_{0}$**and fastest transient parameters**$R_{1}$**,**$\tau_{1}$

Note that this step can be ignored when $R_{0}$ changes slowly with state-of-charge, in which case $R_{0}$ can be identified alongside the other parameters in step 6.

#### Prerequisite parameters

• $v_{\text{oc}}\left(SOC\right)$: interpolant $v_{\text{oc}}\left(SOC\right)$ from step 4.

##### Method

$R_{0}$ is found by numerically fitting a 1RC model to data from the very start of a current pulse, and discarding transient data. As shall be explained, this method improves parameterisation accuracy.

1. Set up the cell model of Eqs. (1) by substituting in the interpolant $v_{\text{oc}}\left(SOC\right)$; use a single RC pair for this step.
2. Select a single current pulse, and extract the portion of the data where I≠0.
3. Say current is switched on at time $t_{0}$; extract the portion of the data between times $t_{0}$ and $t_{0}+\Delta t$, as sketched in Fig. 6.

**Fig. 6:**
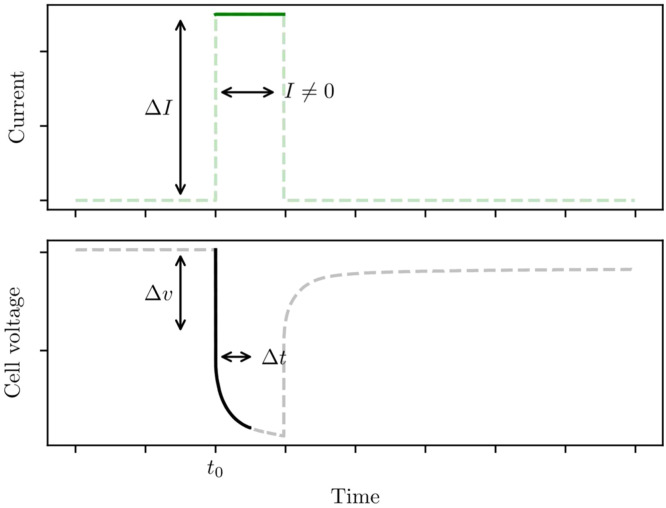
Series resistance $R_{0}$ is found by numerically fitting to the initial transient data, from times $t_{0}$ to $t_{0}+\Delta t$. This is more robust than calculating $R_{0}=\Delta v/\Delta I$. All parameters are fitted when current I≠0, so that they are representative of the active cell.
4. Select an appropriate initial value for parameters $R_{0}$, $R_{1}$, and $\tau_{1}$.
5. Fit $R_{0}$, $R_{1}$, and $\tau_{1}$ to the voltage data v(t) during the selected region:
- assume $R_{0}$, $R_{1}$, and $\tau_{1}$ are constant.
- Numerically integrate Eqs. (1) with parameters $R_{0}$, $R_{1}$, $\tau_{1}$ to get voltage prediction $v_{\text{model}}\left(t;R_{0},R_{1},\tau_{1}\right)$.
- Use a numerical optimiser to solve for

$\underset{R_{0},R_{1},\tau_{1}}{\text{min}}∥v_{\text{model}}\left(t;R_{0},R_{1},\tau_{1}\right)-v\left(t\right){∥}^{2},$subject to the constraint $\tau_{1}^{\text{min}}\leq \tau_{1}\leq \tau_{1}^{\text{max}}$.

6. Record the identified $R_{0}$.
- Note the state-of-charge at the beginning of the selected data-window $SOC(t_{0})$; the cell temperature for the given experiment, $T_{\text{test}}$; and $R_{0}$, the fitted series resistance.
- Record the tuple $\left(R_{0},SOC\left(t_{0}\right),T_{\text{test}}\right)$, which gives the series resistance at a given temperature and state-of-charge.
- Discard $R_{1}$ and $\tau_{1}$.
7. Repeat for all pulse-sets in the discharge and charge data, to form separate discharge and charge datasets of $R_{0}$ at different states of charge.
8. Where relevant, repeat again for each tested cell temperature $T_{\text{test}}$ and pulse-current $I_{\text{pulse}}$.
9. Assemble separate interpolants for $R_{0}\left(SOC,T,I\right)$ in discharge and charge.

An initial parameter estimate must be provided to a numerical optimiser; two choices are available here. If a previous pulse has already been parameterised, its parameters can be used as the initial guess for this step. This should be handled automatically by the parameterisation script. When no previous parameters are available for the initial guess, domain knowledge or trial-and-error should be used. For timescale $\tau_{1}$, trial-and-error can begin at $(\tau_{1}^{\text{min}}+\tau_{1}^{\text{max}})/2$ - the mid-point of the timescale constraints. Sensible estimates of resistance can be obtained from the overpotential.

• ***Common issue:*** poor initial parameter guesses.
• ***Solution:*** spend time and care choosing a good initial guess for the parameters. An optimiser will struggle to find a good parameter set if it is initialised from a bad estimate.

##### Hyperparameters

• Δt: time-window of data to fit to.
• $\tau_{1}^{\text{min}}$: bound, lowest value $\tau_{1}$ can take.
• $\tau_{1}^{\text{max}}$: bound, largest value $\tau_{1}$ can take, chosen from length of available data Δt, and not the length of the overall pulse.

• ***Common issue:*** using overly tight parameter bounds.
• ***Solution:*** use the widest, least constraining parameter bounds possible, as explained in Section 2.2. Better fitting is almost always achieved by using a better initial parameter guess-not by tightening the bound constraints.

##### Explanation

This multi-step parameterisation is a novel aspect of the method, and can prove particularly valuable for long pulses where state-of-charge changes significantly, as well as at state-of-charge extremes, and for cell chemistries where the resistance changes a notable amount in each current-pulse. As explained further in the next step, the change in resistance across a pulse will cause small errors in the parameters. This step extracts series resistance from the beginning of a pulse, which allows subsequent errors to be reduced by giving a model of how resistance changes across a pulse.

The aim is to identify a series resistance $R_{0}$ from the beginning of each pulse. This could be calculated as ΔV/ΔI, where ΔV and ΔI are instantaneous changes in voltage and current, and this provides a good initial guess. However the optimisation method is preferred. Since sampling frequencies are finite, ΔV will contain a mixture of the true series resistance, and rapid transients. Some of these transients will continue to change over the next timepoints, but fast transients cannot always be resolved (for example, it is unrealistic to try fitting transients with 1 s timescales using data sampled at 1 Hz). These fast transients would be ignored when calculating an instantaneous ΔV/ΔI, whereas they are automatically be captured in $R_{0}$ when using the optimisation approach, which leads to better model accuracy. The first transient pair $R_{1},\tau_{1}$ must also be fitted to do this, however these data are discarded, and will be fitted with higher accuracy in step 6. An identical approach can be applied for multi-step parameterisation of each RC pair.

Constraints must be applied to find sensible values for $\tau_{1}$. As noted, it is unrealistic to fit a fast timescale from low sample-rate data, so a lower-bound $\tau_{1}^{\text{min}}\leq \tau_{1}$ is used. Likewise, it is not possible to accurately fit very slow timescales from short data windows, so an upper bound $\tau_{1}\leq \tau_{1}^{\text{max}}$ is also applied. Section 2.2 explains how to choose the hyperparameters Δt, $\tau_{1}^{\text{min}}$, and $\tau_{1}^{\text{max}}$.

A range of constrained numerical optimisation methods can be chosen. Gradient-based methods are typically fast and accurate for this problem. The fitting problem can be insensitive to $\tau_{1}$, meaning a range of timescales give similar results, so gradient-free methods such as Nelder-Mead or particle-swarm optimisation can also be chosen to thoroughly explore the parameter space. Recommended best practice is to try a range of different gradient-based and gradient-free optimisers for every pulse, and choose the solution with the lowest fitting error.

#### **Step 6: Fit transients, given**$R_{0}\left(SOC\right)$**and**$v_{\text{oc}}\left(SOC\right)$

• ***Common issue:*** fitting to both the pulse and rest periods.
• ***Solution:*** only fit $R_{0}$, $R_{i}$, $\tau_{i}$ to data where I≠0; do not include rest periods.

#### Prerequisite parameters

• $v_{\text{oc}}\left(SOC\right)$: interpolant from step 4.
• $R_{0}\left(SOC\right)$: interpolant from step 5.

##### Method

The previously found $v_{\text{oc}}\left(SOC\right)$ and $R_{0}\left(SOC\right)$ are used to construct a base model, and RC resistances and timescales are numerically identified for each individual pulse.

1. Set up the cell model of Eqs. (1) using the previously found interpolants $v_{\text{oc}}\left(SOC\right)$ and $R_{0}\left(SOC\right)$.
2. Select a single pulse, and extract the portion of the data where I≠0.
3. Fit RC parameters $R_{i}$ and $\tau_{i}$ (i=1,…,n) to the selected voltage data v(t), using the same approach as in step 5:
- assume $R_{i}$ and $C_{i}$ are constant throughout the duration of the pulse.
- Use a numerical optimiser to solve for

$\underset{R_{i},\tau_{i}}{\text{min}}∥v_{\text{model}}\left(t;R_{i},\tau_{i}\right)-v\left(t\right){∥}^{2},$subject to the constraints $\tau_{i}^{\text{min}}\leq \tau_{i}\leq \tau_{i}^{\text{max}}$.

4. Find the average state-of-charge across the pulse, and record as $\bar{SOC}$. This can be found as the average of the start and end state-of-charge, since a constant current is used for the pulse.
5. Record the identified parameter sample $\left(R_{i},\tau_{i},\bar{SOC},T_{\text{test}}\right)$.
6. Repeat for all pulse-sets in the discharge and charge data, to form separate discharge and charge datasets.
7. Where relevant, repeat across each tested cell temperature and pulse current.

##### Hyperparameters

• $\tau_{i}^{\text{min}}$: lower bound on timescale $\tau_{i}$.
• $\tau_{i}^{\text{max}}$: upper bound on timescale $\tau_{i}$.
• n: number of RC pairs to fit.

##### Explanation

Voltage transients behave differently when I=0 and I≠0. As a result, parameters are only fitted to data where I≠0, so that they are as accurate as possible for a cell under load. This differs from the usual approaches in the literature where parameters are fitted to both pulses and relaxations (see eg. the ‘layered method’ [7]). The approach of fitting to pulses and relaxations gives a model that is accurate for neither the pulse nor the relaxation. In contrast, fitting with I≠0 gives high accuracy for a cell under load, at the expense of reduced accuracy for the subsequent relaxation to open-circuit voltage. This is a worthwhile trade-off for most applications.

**Fig. 7:**
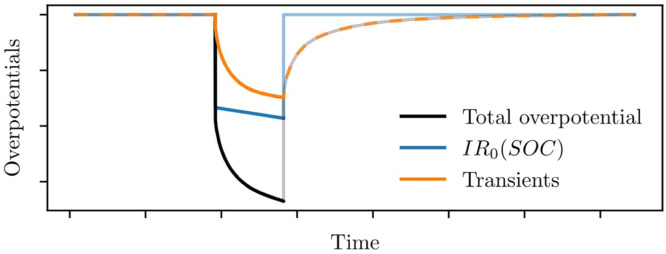
Transients can be identified more accurately by incorporating $v_{oc}$ and $R_{0}$ interpolants from previous steps, since $v_{oc}\left(SOC\right)$ and $R_{0}\left(SOC\right)$ will change as SOC changes. Contributions to the overall overpotential are sketched here using linear interpolation for $v_{oc}\left(SOC\right)$ and $R_{0}\left(SOC\right)$.

State-of-charge will be different at the start and end of a pulse, so $v_{\text{oc}}\left(SOC\right)$ and $R_{0}\left(SOC\right)$ will also have changed. To account for this, the $v_{\text{oc}}$ and $R_{0}$ samples from previous steps are built into functional parameters and incorporated into the model. As shown in Fig. 7, this allows the cell voltage to be more accurately decomposed into open-circuit voltage, series resistance overpotential, and transients. For the same reason, the transients parameters were discarded in step 5, and re-fitted here; the resulting parameter accuracy will improve, as changes in $R_{0}$ have now been accounted for.

Fitted parameters can be interpreted as the average parameter value across the state-of-charge range. Hence, they are assigned to the average state-of-charge of that pulse. This approximation is accurate to leading-order, and becomes increasingly accurate with shorter pulses; trade-offs in pulse length are explored in Section 2.2.

#### Step 7: Build interpolations

**Fig. 8:**
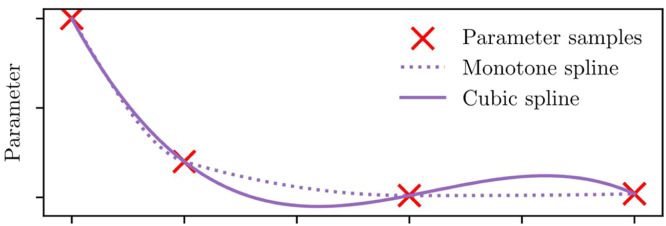
Functional parameters are created by interpolating the fitted parameter samples. The independent variable can be one or more of temperature, state-of-charge, and current. Interpolation methods should be chosen carefully; monotonic interpolators such as linear interpolation, or piecewise cubic Hermite interpolating polynomials (PCHIP, shown here) avoid overshoot, which could otherwise cause problems such as negative resistances.

So far, a set of parameter samples have been produced, which represent each parameter at some given temperature and state of charge. Interpolation is now used, to predict new parameter value between datapoints. Broadly, there are three approaches for doing this. A curve fitting method can be used, which produces parameter estimates that lie close to each parameter sample, to help average out noise. Alternatively, an interpolation can be chosen, which makes sure the parameter functions pass through every parameterised datapoint; a standard or monotonic interpolant could be used for this. Monotonic interpolators ensure the predictions lie within the ranges of adjacent data, which provides a degree of safety since it cannot overshoot. Overshooting is a particular risk with noisy or fast-changing data, and can lead to unwanted results such as negative resistance, as sketched in Fig. 8. Monotonic interpolations avoid these problems, but will also miss any peaks or troughs between datapoints.

Linear interpolation is monotonic, and can be applied in high dimensions. Parameters can be projected onto a grid, which allows for computationally faster linear interpolation, however this has the potential to introduce small errors. Polynomial interpolation is a common non-monotonic interpolation, but should be strongly avoided due to overshooting. Splines are a good choice for a non-monotonic interpolator, with cubic splines being the typical choice. Splines can also be used in a fitting setup; since they are not constrained to pass through every datapoint, they can help filter out parameter noise. A well-designed fitting method needs to balance goodness-of-fit against overall complexity.

Monotonic interpolation is always suitable for $v_{\text{oc}}\left(SOC\right)$ since open-circuit voltage always increases with state-of-charge. For the remaining parameters, recommended best practice is to try curve fitting, standard interpolation, and monotonic interpolation, and examine the results for benefits such as smoothing; errors such as missed peaks; and problems such as negative resistance. Practitioners should choose interpolation methods with care, and make sure to validate the results on unseen data. Generally speaking, smooth monotonic interpolation is preferred for clean parameter sets, and smoothing splines for noisier parameters. As noted, timescales $\tau_{i}$ should be used in place of capacitances $C_{i}$ when modelling, and therefore interpolations should be built over $\tau_{i}$ and not $C_{i}$.

A model will only be accurate within the range of parameterisation data. For example, it will not be accurate at a low state-of-charge or high temperature if there are no parameterisation pulses there. Therefore, it is computationally convenient to choose an interpolant that does not extrapolate, so that the model is never used outside the range of available data. However, this can cause numerical issues - a differential equation solver may well explore SOC>1 during a numerical differentiation step, and a ban on extrapolation would cause the solver to fail here. This can be avoided by adding fictitious datapoints at either side of the domain. For example, a set of carefully chosen fictitious datapoints could be included at SOC=1.01. This means the interpolation can still be banned from automatically extrapolating, but a small, carefully controlled extrapolation has been made to improve the numerical robustness. It is important that a model implementation decides whether to prioritise robust numerics, avoiding parameter extrapolation, or both; the most appropriate choice will depend on the model use-case.

#### Method validation

The validations presented here are based on data generated in [4], for which this method was developed. Following [4], the model is fitted to discharge data taken at a single test-current, though it could equally be used for modelling in charge, or for producing current-dependent parameters. Numerics are implemented in Python, using the PyBOP package [11]. Scripts for repeating this validation are available on GitHub at https://github.com/MarkBlyth/parameterisation_methodsx, and archived through Zenodo at https://doi.org/10.5281/zenodo.19072964. While the scripts are set up for use with a Neware battery cycler, tools such as PyProBE [12] can be used to handle data from other cyclers. Similarly, the scripts use appropriate hyperparameters and parameter guesses for the tested cell, however these can easily be changed for different test cells.

Experiments are performed on Graphite / NMC 622 pouch cells, manufactured in a single 250-cell batch by LiFun. Cell capacity is taken as 2.2 Ah, based on capacity throughput at 40 ${}^{\circ }$C. Steps 5 and 6 are combined, so that all parameters are found in a single optimisation step. Parameterisation data are made up of 24 current pulses^2^; the final three pulses are ignored, as the cell reaches lower voltage cutoff during these. Details of the parameterisation are presented in Table 1. Parameter identification is performed on each discharge pulse using a range of optimisers, and the best result is chosen each time. Table 2 shows which optimisers were used, and how many pulses each optimiser did the best on. As expected, gradient-based optimisers perform well for this problem. Table 2

**Table 1:** Choice of hyperparameters for the method validation.

Hyperparameter	Description	Value
$T_{\text{test}}$	Test temperature [${}^{\circ }$C]	25
$I_{\text{pulse}}$	Pulse current [A]	1.8
$t_{\text{pulse}}$	Pulse time [s]	180
$t_{\text{settle}}$	Settle-time [h]	2
n	Number of RC pairs	2
$\tau_{1}^{\text{min}}$	First timescale lower-bound [s]	5
$\tau_{1}^{\text{max}}$	Second timescale lower-bound [s]	5
$\tau_{2}^{\text{min}}$	First timescale upper-bound [s]	180
$\tau_{2}^{\text{max}}$	Second timescale upper-bound [s]	180
Δt	$R_{0}$ window [s]	N/A

**Table 2:** List of optimisers used. Every optimiser is applied to every pulse. ‘Number of successes’ shows how many pulses each optimiser performed best on. Gradient-based methods are seen to perform well for this problem.

Name	Type	Number of successes
SciPy SLSQP	Gradient-based	6
SciPy trust-constr	Gradient-based	15
Particle swarm optimisation	Gradient-free	0
CMAES	Natural evolution	0
SNES	Natural evolution	0
XNES	Natural evolution	0

A detailed analysis is given for the results of the 25 ${}^{\circ }$C parameterisation. Other temperatures are repeated to produce the temperature-dependent model, however these all follow the same procedure as for 25 ${}^{\circ }$C, so a full account is not necessary. The resulting model, parameters, data, and scripts are all freely available alongside the provided parameterisation scripts.

**Fig. 9:**
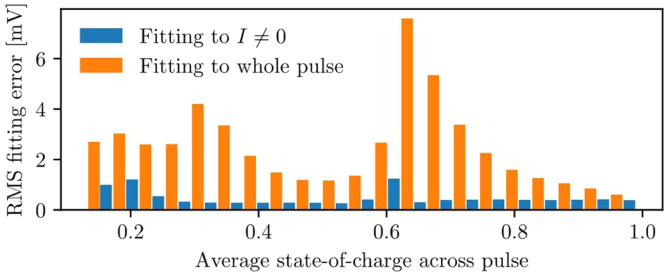
Fitting error for each discharge pulse in the parameterisation experiment. Blue bars show the performance of the method described here, where parameter fitting is performed when I≠0. For comparison, orange bars show the fitting error when parameters are fitted to the entire pulse (consisting of both the current-draw and relaxation periods) using an otherwise identical method. Fitting to I≠0 shows superior accuracy, and accurate parameter fits are achieved across the full state-of-charge range.

**Fig. 10:**
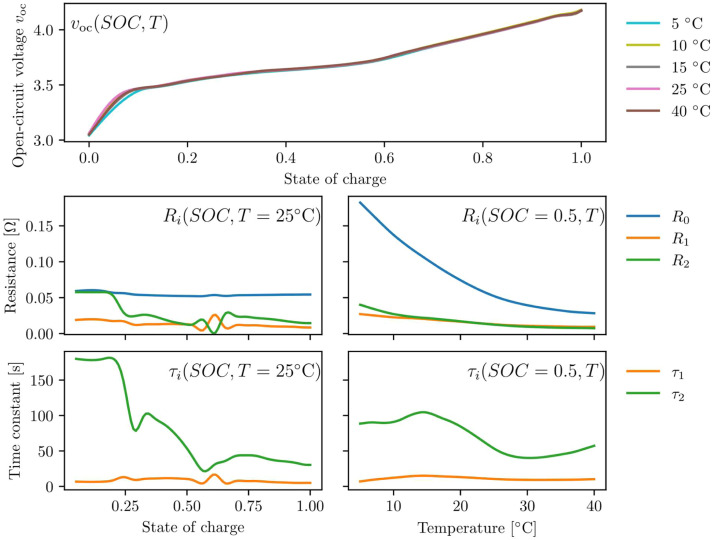
identified parameters, using a Clough-Tocher 2d monotonic interpolation. Each parameter is shown as a function of state-of-charge at 25°C, and as a function of temperature at 50 % state-of-charge.

Each pulse incurs a fitting error due to imperfections in the model, such as the assumption that parameters are constant across the pulse. Fig. 9 shows the final fitting error for each parameterised pulse. A low fitting error is achieved, which shows that the parameterisation method works well. Notably, the conventional approach of fitting parameters to both the pulse and relaxation causes a substantially larger fitting error than the method recommended here, motivating the parameterisation strategy introduced in this work. A slightly higher model error occurs at low state-of-charge, where parameters change rapidly throughout a pulse. In both cases, error increases slightly at 60 % state-of-charge, which is possibly driven by behaviour changes during a phase transition in the electrode materials; the same effects have been found in other works [15]. Fig. 10 shows the identified model parameters, where the timescales and RC resistances are seen to change in this region.

**Fig. 11:**
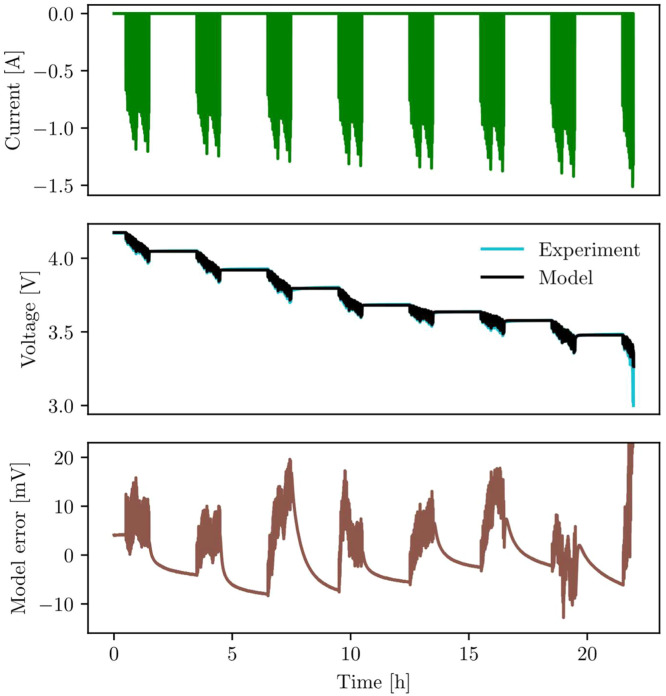
Model validation on a rapidly varying power-based drive cycle. The model generally agrees well with experimental data, except at the bottom of the state-of-charge range where the error rapidly increases. Model error peaks at 262 mV at the end of the test, which goes substantially beyond the chosen axis range.

Models should be validated on a different data-set to the fitting data. Fig. 11 compares model and experimental data on a drive-cycle with highly variable currents, representative of the power demands on an electric vehicle. Notably, the currents throughout the drive cycle are different to those used for parameterisation; this will increase the model error, but offers a useful test of how well the model performs on unseen data. A capacity of 2.13 Ah is used in the model, which reflects capacity losses in the cell after the parameterisation and before the validation experiments. The model generally performs well, and model errors compare favourably to literature results [20].

#### Limitations

For some cell chemistries, open-circuit voltages are different in charge and discharge, with hysteresis coming from phase changes in the electrode materials. Timescales and resistances can also differ between charge and discharge. This method handles these differences by building separate discharge and charge parameter sets, and does not attempt to build or parameterise a model for switching between the charge and discharge regimes. Users may therefore choose to average out the two parameter sets; to use one set for discharge models and a separate set for charge models; or to design a sub-model that switches between the discharge and charge parameters as appropriate. The most appropriate choice will depend on how the final model is to be used.

RC pairs capture transient effects in the cell voltage. The timescales of these transients are different for a cell under load (I≠0) versus a cell returning to open-circuit voltage (I=0). Many methods in the literature ignore this distinction, and lose accuracy across all usage cases, as shown in Fig. 9. This method fits parameters only when a cell is under load (I≠0), so that parameters are fully representative of how a cell behaves in use. It would be simple to modify this method to solve for parameters and initial conditions when I=0. However, changing between parameter sets at I≠0 and I=0 introduces a switching regime into the model, and as a result, model states would need to change across the switch-boundary to ensure that transient voltages are handled correctly. It is challenging to design a model that rigorously addresses the switching, and this remains an important research question.

The Thevenin model describes overpotentials using resistors, which suggests overpotential varies linearly with current. However, overpotentials of a real cell depend nonlinearly on current. This means the circuit parameters should depend on cell current, and should also differ between charge and discharge. This method handles current-dependency by allowing the user to build multiple parameter sets for different C-rates, in which case cell current becomes a dependent variable in the interpolated functional parameters. Alternatively, users may select a single representative current for all tests, in which case the model will see a small loss of accuracy for other cell currents. This approach is used in successfully Section 3, and for many applications, the accuracy loss is small enough to be acceptable.

Models lose accuracy at low and high state-of-charge, as it is not possible to fully cover the voltage cutoff regions with a current pulse. Instead, careful extrapolation must be used.

Pulse-parameterisation methods are constrained by a trade-off on pulse duration. Longer pulses have the disadvantage of fewer parameter samples, and each sample is averaged over a larger state-of-charge range. Shorter pulses typically give more accurate parameters, however each pulse needs to be long enough to accurately fit transients from. A possible workaround is to parameterise from multiple datasets, each containing different pulse lengths. Alternatively, discretisation-based approaches such as splines could be used to directly solve for functional parameters. Accuracy would be improved as the parameters can then change throughout a pulse; nevertheless, this approach may have identifiability issues. A thermal model could also be included to address cell heating during longer pulses. Thermal and electrical models can be created in tandem, using a thermal extension of the method presented here [4], though parameterising a cell with a varying temperature remains an open challenge.

## Ethics statements

This work does not involve studies with animals or humans.

## Supplementary material and/or additional information

All data and code are available on GitHub at https://github.com/MarkBlyth/parameterisation_methodsx, and archived with Zenodo at https://doi.org/10.5281/zenodo.19072964.

## Specifications table

**Table:** 

**Subject area**	Engineering
**More specific subject area**	Battery modelling
**Name of your method**	Method for time-domain parameterisation of an empirical battery model
**Name and reference of original method**	Blyth, Mark, and Alastair Hales. "Thermal parameters fitted from electrical data enable lumped models of heterogeneous battery cells by predicting their effective temperature." *Journal of Energy Storage* 140 (2025): 118,856.
**Resource availability**	The provided data were obtained using the following setup: • Battery cycler: Neware 5 V 6A cycler • Cell thermal control: custom Peltier feedback controllers • Cells: 1.8 Ah NMC 622 pouch cells, manufactured by LiFun • Demonstration scripts implemented in Python *All data and code are available on GitHub at https://github.com/MarkBlyth/parameterisation_methodsx, and archived with Zenodo at*https://doi.org/10.5281/zenodo.19072964.

## CRediT authorship contribution statement

**Mark Blyth:** Conceptualization, Methodology, Software, Validation, Investigation, Formal analysis, Data curation, Writing – original draft, Visualization. **Amey Gupta:** Writing – original draft, Writing – review & editing. **Alastair Hales:** Conceptualization, Resources, Writing – review & editing, Supervision, Project administration, Funding acquisition.

## Declaration of competing interest

The authors declare that they have no known competing financial interests or personal relationships that could have appeared to influence the work reported in this paper.

## Data Availability

All data and code are available on GitHub at https://github.com/MarkBlyth/parameterisation_methodsx, and archived through Zenodo at https://doi.org/10.5281/zenodo.19072964.

## Footnotes

1 While this helps prevent issues, timescales and resistances will still be of a different order of magnitude. There are two options to further improve this. First, a scale-factor can be inserted onto $R_{i}$ or $\tau_{i}$ between the model and the optimiser, which can be applied automatically in PyBOP [11]. Alternatively, the model can be defined in terms of conductances $g_{i}=1/R_{i}$, which will be of the same order of magnitude as the timescales. The authors have not found any extra benefit in using these tricks, however they may come in useful for particularly difficult parameterisation problems.

2 This differs depending on the test-temperature, as hotter cells have a larger accessible capacity than colder cells, so can undergo more pulses before reaching lower voltage cutoff.
